# Adverse Effects of Methylmercury: Environmental Health Research Implications

**DOI:** 10.1289/ehp.0901757

**Published:** 2010-06-08

**Authors:** Philippe Grandjean, Hiroshi Satoh, Katsuyuki Murata, Komyo Eto

**Affiliations:** 1 Department of Environmental Medicine, University of Southern Denmark, Odense, Denmark; 2 Department of Environmental Health, Harvard School of Public Health, Boston, Massachusetts, USA; 3 Department of Environmental Health Sciences, Tohoku University Graduate School of Medicine, Sendai, Japan; 4 Division of Environmental Health Sciences, Akita University, Akita, Japan; 5 National Institute for Minamata Disease, Minamata, Japan

**Keywords:** empirical research, environmental exposure, epidemiology, methylmercury compounds, prevention and control, public policy, seafood, toxicology

## Abstract

**Background:**

The scientific discoveries of health risks resulting from methylmercury exposure began in 1865 describing ataxia, dysarthria, constriction of visual fields, impaired hearing, and sensory disturbance as symptoms of fatal methylmercury poisoning.

**Objective:**

Our aim was to examine how knowledge and consensus on methylmercury toxicity have developed in order to identify problems of wider concern in research.

**Data sources and extraction:**

We tracked key publications that reflected new insights into human methylmercury toxicity. From this evidence, we identified possible caveats of potential significance for environmental health research in general.

**Synthesis:**

At first, methylmercury research was impaired by inappropriate attention to narrow case definitions and uncertain chemical speciation. It also ignored the link between ecotoxicity and human toxicity. As a result, serious delays affected the recognition of methylmercury as a cause of serious human poisonings in Minamata, Japan. Developmental neurotoxicity was first reported in 1952, but despite accumulating evidence, the vulnerability of the developing nervous system was not taken into account in risk assessment internationally until approximately 50 years later. Imprecision in exposure assessment and other forms of uncertainty tended to cause an underestimation of methylmercury toxicity and repeatedly led to calls for more research rather than prevention.

**Conclusions:**

Coupled with legal and political rigidity that demanded convincing documentation before considering prevention and compensation, types of uncertainty that are common in environmental research delayed the scientific consensus and were used as an excuse for deferring corrective action. Symptoms of methylmercury toxicity, such as tunnel vision, forgetfulness, and lack of coordination, also seemed to affect environmental health research and its interpretation.

Although the toxicology and environmental epidemiology of methylmercury have been recently outlined [Clarkson and Magos 2006; Grandjean et al. 2005; United Nations Environmental Programme (UNEP) 2002], the sequence of scientific discoveries and consensus building reveals important caveats and complications that may have a wider relevance to environmental health research.

Metallic mercury and its inorganic salts have been known since antiquity, but organic mercury compounds with a covalent bond between the mercuric ion and the organic radical were first described in the 19th century. The toxic actions became readily apparent in laboratory accidents, and the description of the clinical syndrome noted the “unique character of their symptoms, which do not resemble those produced by any known disease” (Edwards 1865). The clinical picture included sensory disturbance of the lower legs, lower arms, and face; visual field constriction (“tunnel vision”); deafness, ataxia; and dysarthria. This seminal publication became widely known at first but was later forgotten. Major events in the subsequent environmental history of methylmercury are listed in Table 1.

In a commentary on the regulatory delays in dealing with methylmercury poisoning in Minamata, Japan, Jun Ui [as quoted by D’Itri and D’Itri (1978)] wrote:

It might be a coincidence, but a strange, parallel relationship was observed between the actual symptoms of Minamata Disease and the reactions of these formal organizations. A constriction of the visual field was common among all organizations. Ataxia, a loss of coordination between various parts of the body, was often exhibited in contradictions between the measures taken by various parts of the government. There was also a loss of sensation as the appeal of the victims went unheard and there was little effort to grasp the situation as a whole. Many organizations also reacted with spasmic convulsions when they faced the problem. This was followed by mental retardation and forgetfulness.

In the present review we consider the extent to which such manifestations also affected the performing, reporting, and recognition of environmental methylmercury research.

## Early Evidence of Human Toxicity

Despite Edwards’s alarming report on fatal methylmercury poisonings (Edwards 1865), this substance was applied in the search for a cure for syphilis. Not surprisingly, the experimental treatments resulted in severe side effects in the patients (Hepp 1887); therefore, this approach was not further pursued. However, the microbial toxicity was used in applications of methylmercury as a fungicide, which became important commercially starting around 1914, initially with few published records of adverse effects (Franke and Lundgren 1956; Hunter and Russell 1954). This application of mercury became widely used in developing countries as part of the “green revolution,” without any monitoring of dissemination of mercury in the environment or of associated adverse effects.

With additional clinical cases being reported, the unique combination of signs and symptoms became established as a key to the diagnosis of fully developed methylmercury poisoning. A postmortem examination of a deceased worker showed damage to the cerebral and cerebellar cortices that corresponded to the patient’s neurological signs (Hunter and Russell 1954).

Expanded use of mercury fungicides and improper labeling paved the way for a series of food poisoning incidents during famines in several countries, where treated seed grain was mistakenly used for bread making. The first cases were reported in Iraq in 1955–1956 and 1959–1960 (Jalili and Abbasi 1961), then in Pakistan in 1961 (Haq 1963), in Guatemala in 1965 (Ordonez et al. 1966), and again in Iraq in 1970–1971 (Bakir et al. 1973). Both methylmercury and related ethylmercury compounds had been used for seed dressing. Large numbers of poisonings and deaths occurred, but the emergency circumstances during a famine made data collection difficult, with limited opportunities to record the extent of the exposures.

The most detailed studies of 93 poisoned adults in Iraq identified facial paresthesia as the earliest clinical sign of poisoning, with a clear dose dependence (Bakir et al. 1973). Official records acknowledged that 6,530 patients were hospitalized and 459 died (Bakir et al. 1973), but the amount of treated grain used (100,000 tons) would suggest that many more could have been poisoned, although circumstances did not allow follow-up of a representative group of subjects. The first author of the 1973 *Science* report (Bakir et al. 1973), Farhan Bakir, was later recognized as Saddam Hussein’s personal physician, now in exile along with at least one other Iraqi coauthor (Giles 2003; Hightower 2009). Although any error or bias in the research reports is difficult to determine today, one can assume that methylmercury toxicity was unlikely to have been exaggerated.

Bakir et al.’s (1973) dose–response data appeared to confirm a previous risk assessment of methylmercury, as determined by the Joint Expert Committee on Food Additives (JECFA) under the World Health Organization and the Food and Agriculture Organization of the United Nations (JECFA 1978). These conclusions formed the basis for risk assessment for the next 25 years (JECFA 2003).

## Unexpected Exposure Pathways

On 1 May 1956, Hajime Hosokawa and Kaneki Noda submitted a report to the Minamata Health Centre in Japan on a mysterious series of four cases of the same, unknown neurological disease (Social Scientific Study Group on Minamata Disease 1999). A few weeks later, a committee of medical experts found an additional 30 patients along the shores of Minamata Bay, among whom the first cases apparently developed as early as 1953.

A genetic cause of the disease was soon ruled out because it occurred in unrelated subjects (Nagano et al. 1957). However, the pattern of disease occurrence resembled that of an infectious agent. In one severe instance, 8 of 11 family members were afflicted, and the remaining 3 subsequently also appealed for recognition. Most of the patients were fishermen and their families who resided near the coast. Additionally, a mysterious dancing disease had recently emerged in fishermen’s cats (Social Scientific Study Group on Minamata Disease 1999). The experts suspected that a toxic metal had contaminated the seafood. On 4 November 1956, the day after the release of this report, the prefectural authorities wisely announced a warning against eating seafood from Minamata Bay. However, a ban against eating seafood from Minamata Bay was not approved by the Ministry of Health and Welfare in Tokyo because of uncertainty regarding involvement of seafood in general in causing the disease (Harada 2004; Social Scientific Study Group on Minamata Disease 1999).

During the next 2 years, several studies failed to pinpoint the exact cause of the seafood-associated disease, and the available information caused some confusion. For example, the Japan Chemical Industry Association claimed that the disease could be due to leakage of explosives dumped during World War II (Mishima 1992). Manganese was suspected for a while, as were selenium, thallium, and copper. A summary of these findings, along with personal observations, was published in *Lancet* (McAlpine and Araki 1958). These authors mentioned that metal poisoning was suspected and briefly reviewed methylmercury (citing the report by Hunter and Russell 1954) along with other neuro-toxicants. The first international report on methylmercury as a likely cause of Minamata disease was published soon thereafter (Kurland et al. 1960). The source of methylmercury was later found to be an acetaldehyde plant owned by the Chisso Company, which used mercury as a catalyst.

In hindsight, it seems strange that methylmercury was not recognized right away as the most likely cause of Minamata disease, given the descriptions by Edwards (1865) and Hunter et al. (1940) of its unique clinical features. The similarity in clinical symptoms was in fact noted (Tokuomi et al. 1960), as were the pathology findings from autopsies of deceased victims (Matsumoto 1961; Takeuchi et al. 1959, 1960). However, thin-layer chromatography identification of methylmercury was not successful until 1962, when the substance was identified in sludge from the acetaldehyde plant and the bottom sediment of the effluent channel (Irukayama et al. 1962). Elevated methylmercury concentrations were subsequently documented in seafood and in tissues of deceased patients. It seemed highly unlikely that this exotic substance caused the contamination of the seafood from Minamata Bay and surrounding waters: How could an expensive mercury fungicide be the cause of the poisonings? Methylmercury was eventually acknowledged by governmental authorities in 1968 to be the cause of Minamata disease (Social Scientific Study Group on Minamata Disease 1999).

In the meantime, new cases of Minamata disease had been discovered in Niigata, Japan, in 1965 on the main island of Honshu (Tsubaki et al. 1969, 1977); methylmercury releases originated from the same production processes using mercury as a catalyst. Now the researchers were better prepared, and analytical methods were readily available to apply to environmental samples, tissues, and hair. Because the Niigata cases in general were milder, the studies provided useful insights into less pronounced cases of Minamata disease. Further studies during the 1970s and 1980s identified a variety of delayed symptoms in people exposed to methylmercury (Kinjo et al. 1993), and the adverse effects of methylmercury pollution were documented in many communities around the Shiranui Sea, some of them a substantial distance from Minamata (Ninomiya et al. 1995; Yorifuji et al. 2008).

Decades earlier, it had been discovered that methylmercury could be spontaneously formed from inorganic mercury employed in acetaldehyde production (Vogt and Nieuwland 1921; Zangger 1930), and in fact, cases of methylmercury poisoning occurred in German acetaldehyde production workers (Koelsch 1937). The two factories in Minamata and Niigata had copied the German production process, but the toxicity reports went unnoticed for > 50 years (Ishihara 2002).

## Diagnostic Difficulties

Despite the characteristic features of severe methylmercury poisoning, linkage to a causative exposure could be complicated by the latency period of several weeks to months between the exposure and the development of clinical symptoms (Edwards 1865; Franke and Lundgren 1956). In addition, early symptoms, as seen in farmers and factory workers, were hard to recognize. In the words of Ahlmark (1948), “Such symptoms [of methylmercury poisoning] scarcely differ from those generally found in neurasthenics when they think that they have been exposed to toxic risks.” In fact, one worker was thought to suffer from hysteria and underwent electroshock treatment (which did not help) before being diagnosed with methylmercury poisoning (Herner 1945).

In Japan in 1977, the Environment Agency issued a notice on certification of patients, and the criteria for Minamata disease diagnosis became the object of much discussion and legal proceedings (Harada 2004). The legal requirement to identify bona fide cases of Minamata disease and to separate this diagnosis from other abnormalities unrelated to such exposure required considerable medical attention and resources. Meanwhile, increasing numbers of likely victims were being discovered, in part due to the continued pollution and environmental dissemination. A court decision in 2004 provided recognition and compensation to many additional Minamata disease patients (most of whom had died by that time) (Ushijima et al 2010). However, many thousands were thought to be affected, although not to a degree justifying compensation based on the existing case criteria. Only in 2009 was a law enacted to provide compensation to most of the remaining groups of victims (Martin 2009).

Part of the inertia was probably due to previous embarrassments caused by having to retract mistaken conclusions of earlier suspected causes, in combination with legal and political rigidity. The resistance and lack of cooperation from Chisso were also an important factor. Most embarrassing, toxicity experiments were carried out in the late 1950s by Chisso’s company doctor, Hajime Hosokawa. Ten cats were fed standard cat food mixed with effluent from the acetaldehyde plant, where mercury was used as a catalyst (Eto et al. 2001). At that time, the researchers were not aware that the effluent contained methylmercury. The exposed cats developed symptoms similar to those seen in cats that had eaten fish from the bay. Only in 1969 did Hosokawa reveal that the results existed and had been suppressed by his employer. A detailed scientific account was eventually published after a 40-year delay (Eto et al. 2001).

Even at a hearing in 1971, a representative from Chisso, Keiji Higashidaira, still claimed that Minamata disease was due to rotten fish and not mercury contamination from the factory (Mishima 1992). Thus, the company continued to claim innocence and lack of proof for many years. Only after legal defeat did Chisso formally agree to pay compensation to the victims.

Similar problems occurred elsewhere. Among the best documented cases is the serious mercury contamination of the Kenora area in Ontario, Canada. Beginning in 1962, a chloralkali plant released mercury waste into a local lake, and pulp wastes were released from a nearby paper production plant that used phenylmercury in slimicides. Contamination of freshwater fish affected the livelihood and health of bands of Ojibway people and sportsfishers. But the challenge of “show me someone who had died of mercury poisoning” became an oxymoron, because autopsies were not conducted on the exposed Ojibway, and their blood mercury concentrations were kept secret (D’Itri and D’Itri 1978; Wheatley et al. 1979).

In the United States, Korns (1972) reported the case of a housewife who had eaten swordfish daily trying to lose weight; she had developed blurred vision, fatigue, ataxia, and headaches. She was under psychiatric treatment for psychosomatic disease until methylmercury toxicity was recognized. More cases among dieters emerged (Genuis 2009), and a series of affected patients among avid sushi eaters was identified by a practicing specialist in California (Hightower 2009).

## Developmental Susceptibility

A new era in methylmercury toxicology was heralded by the first description of congenital methylmercury poisoning in 1952 (Engleson and Herner 1952). A Swedish family had inadvertently used flour made from methylmercury- treated seed grain. One infant had eaten porridge made with this flour since weaning at 9 months of age. The child’s pregnant mother had also eaten the porridge without suffering any adverse effects herself. After delivery of the second child, both children were found to be mentally retarded and severely deficient in motor development; their condition was virtually unchanged 2 years later. Although the doses received by the mother and her two children are not known (Engleson and Herner 1952), this case report suggested that the nervous system was much more vulnerable to methylmercury toxicity during early development, including the fetal stage.

In Minamata, a series of infants poisoned in their mother’s womb was recorded by the first investigative team from Kumamoto University (Kitamura et al. 1957). The researchers also noted that many children born from 1955 onward suffered from developmental disturbances that suggested diffuse cerebral dysfunction (Harada 2004). Children < 9 years of age appeared to be particularly numerous among the patients. In many cases, the pregnant woman appeared completely healthy, despite carrying a baby who suffered congenital methylmercury poisoning (Takeuchi et al. 1964).

Most of these children were not immediately diagnosed because the spastic paresis syndrome was less distinctive than the clinical picture of the adult poisoning cases. The early signs of congenital poisoning (i.e., mental retardation, movement problems, seizures, primitive reflexes, and speech difficulty) could be mistaken for some other disease and over-looked, especially in mild cases.

In Japan, parents often keep a piece of the umbilical cord from their children as a traditional token of luck. During the 1960s, specimens were collected by Masazumi Harada, who showed that children with recognized congenital Minamata disease had the highest concentrations of methylmercury in preserved umbilical cords, whereas those with “ordinary” mental retardation had levels between those of the poisoned subjects and those of the controls (Akagi et al. 1998; Harada et al. 1977; Nishigaki and Harada 1975).

Neuropathology data were also being compiled because detailed autopsies were followed by histological, histochemical, and chemical examinations. It became clear that the adult disease was associated with localized lesions in certain brain areas (e.g., the calcarine, post-central, precentral, and temporal transverse cortices and deep structures of the cerebellar hemispheres of the brain), along with lesions of peripheral sensory nerve fibers (Takeuchi and Eto 1999). Methylmercury poisoning in children showed more widely distributed damage on the brain. However, infants and children who had been poisoned prenatally (from the mother’s diet) showed a diffuse pattern of damage with disruption to normal structures (Takeuchi 1968; Takeuchi and Eto 1999).

These findings strongly supported the notion that early developmental exposure causes a much more serious disease in children than in individuals exposed as adults. As stated by Harada (1977),

It may thus be supposed that the fetal brain is more fragile and susceptible to toxic agents, since it is immature and still undergoing development. . . .

Clearly, prevention of Minamata disease, especially congenital cases, is a first requirement, and the greatest care should be taken by pregnant women since the fetus has a higher sensitivity.

After the main poisoning incident in Iraq in 1970–1971, the pediatrician Laman Amin-Zaki teamed up with colleagues from the United States to study the effects of methylmercury exposure in 49 children. Although the exposed children were examined by basic neurological tests at various ages, the development of language and motor function of children exposed prenatally was found to be delayed (Amin-Zaki et al. 1978). In a later report Marsh et al. (1987) described the use of advanced analytical technology to determine mercury concentration profiles in single hair strands, so that the researchers could get a calendar record of methylmercury exposure during the entire duration of the pregnancy. These dose measures suggested that a greater vulnerability of the developing nervous system would result in adverse effects at an exposure that was one-fifth of the doses causing adverse effects in adults.

“The occurrence of prenatal intoxication also calls for caution” was the scientific consensus in 1972 (JECFA 1972). Later on, JECFA also recognized that “clinical data from Japan indicate that the fetus is more sensitive than the mother,” although the committee refrained from recommending any special protection (JECFA 1978). The risk assessment was therefore based on toxicity in adults and remained that way for the next 25 years.

## Experimental Evidence

In the early 1900s, inorganic chemistry provided a framework for interpreting the very different properties of methylmercury and inorganic mercury (Grimm 1925; Jensen 1937), although it was not appreciated by toxicologists until much later. The first fatal cases of methylmercury poisoning in humans inspired experimental studies to examine its toxic effects in rats, dogs, cats, rabbits, and one monkey. The common feature was an ascending paralysis accompanied by movement difficulties, tremors, blindness, disturbance in hearing, and irascibility in the animals (Hepp 1887; Hunter and Russell 1954). These results were in excellent accordance with the clinical appearance of human poisoning cases. Hunter and Russell (1954) also demonstrated lesions in relevant brain cells and regions as the likely basis of the clinical manifestations. A crucial observation was that cats given methylmercury and related organic mercury compounds showed the same symptoms as cats that had succumbed to eating seafood from Minamata Bay (Eto et al. 2001; Sebe et al. 1961; Takeuchi et al. 1960). Although entirely different from those caused by mercury vapor or inorganic mercury compounds, species differences in vulnerability complicated the evaluation, and only recently did the common marmoset emerge as the best laboratory model of human methylmercury neurotoxicity (Eto et al. 2002).

An important new insight emerged when delayed effects of developmental neurotoxicity were reported in experimental animals in 1972. The key finding was that rats exposed during early development showed adverse effects that at first were not apparent, but later on became obvious as deranged behavior in the mature animals (Spyker et al. 1972). For the first time these experimental results confirmed the increased sensitivity of the brain during development, in support of the Swedish report 20 years earlier (Engleson and Herner 1952) as well as the Minamata evidence. However, at a federal court hearing in the United States, the first author of the Spyker et al. (1972) study testified and presented a movie showing abnormal behavior in prenatally exposed offspring that had first appeared perfectly normal; the judge expressed surprise that disabilities in mice had anything to do with human beings and questioned whether the abnormalities could constitute a reason to regulate mercury (Cranmer J, personal communication).

More recent toxicological studies have aimed at identifying toxic mechanisms and vulnerable time windows especially in relation to brain development (Clarkson and Magos 2006). Since 1980, when the term “methylmercury compounds” was introduced as a medical subject heading, the U.S. National Library of Medicine has listed > 1,000 publications on experimental toxicology of this substance. At present, methylmercury is one of the environmental pollutants with the most extensive toxicology documentation.

## Wildlife Poisonings

At Minamata, marine organisms such as octopi and sea bass were found floating near the shore starting around 1950, and dying fish could be scooped up by hand. Crows were reported to be becoming sick and dying in the area. By 1953, cats were frequently dying from cramps with a condition dubbed “dancing disease.” Kitamura et al. (1957) reported that 50 of 61 cats bred by families of Minamata disease patients died in 1953–1956. By the mid-1950s, the reports of toxic effects on marine life began to extend to nearby coasts.

At the time when methylmercury poisoning occurred among Japanese fishing populations, the same substance was being widely applied for seed dressing in Sweden and other countries. Along with pollution from the paper industry and chloralkali plants, these processes caused environmental accumulation of methylmercury in food chains.

Predatory and seed-eating birds started to develop overt poisoning, and ornithologists became alarmed (Landell 1968). When the cause of the problem was eventually realized, sea eagles and other bird populations were seriously threatened by widespread environmental mercury contamination. Representatives from agriculture insisted that mercury treatment of seeds could not be discontinued without serious financial losses and that detailed research would be needed to document the extent to which mercury might be dispersed in the environment and contribute to bird mortality (Landell 1968).

In the early 1960s, the concern about mercury toxicity inspired some screening efforts for mercury concentrations in foods and environmental samples by neutron activation analysis. When mercury speciation became possible, biomagnification of methylmercury was documented, with increasing concentrations in aquatic food chains. The highest levels were present in predatory fish and fish-eating birds (ospreys and sea eagles), which also exhibited numerous cases of poisoning and reproductive failure (Borg 1969).

A turn in the debate occurred in 1964, when unused seed grain treated with methylmercury had been used as chicken feed in Sweden (Tejning and Vesterberg 1964). In a small study of two hens and a total of six eggs laid by these hens, a high mercury content of 5 mg/kg was found in one of the eggs. This report spurred a ban in several countries against the import of Swedish eggs. The issue of methylmercury transmission in food chains suddenly became highly relevant both to human health and commerce and therefore attracted regulatory attention.

## Environmental Mercury Methylation

It came as a major surprise that methylmercury can be formed from inorganic salts in the environment, as demonstrated by the simple experiment conducted by Swedish researchers (Jensen and Jernelov 1967), who showed that inorganic mercury could be converted into methylmercury in sediment from a home aquarium. After autoclaving the sediment, no methylmercury was formed, thus suggesting that microorganisms played a role (Jensen and Jernelov 1967). Extended studies documented the concentration dependence as well as the generation of volatile dimethylmercury (Jensen and Jernelov 1969), and other research showed that methylcobalamin (vitamin B_12_) could transfer a methyl group to the mercuric ion nonenzymatically (Wood et al. 1968). These methylation processes were probably of little significance in Minamata and Niigata, where methylmercury was formed in the acetaldehyde plants as part of the catalyst reactions. However, methylation of mercury from all sources causes worldwide contamination of freshwater fish and seafood by methylmercury.

The widespread use of methylmercury for seed dressing, along with other mercury sources such as fungicides used in paper mills, have added to the pollution of waterways and coastal waters. Many rivers and lakes became so polluted with mercury that fish advisories against eating sport fish were issued, especially in countries such as Canada, Sweden, and the United States [U.S. Environmental Protection Agency (EPA) 2009]. Studies in North America verified that biomagnification took place, especially near paper mills and chloralkali plants, once again with the highest concentrations in top carnivores (Fimreite 1974). Although methylmercury contamination of fish had been thought to constitute a local problem in Japan, it now appeared to occur worldwide, with serious ecological effects and dangerous human exposures.

Mercury releases into the aquatic environment could also come from air pollution, for example, from municipal incinerators and power plants burning coal that contains mercury. The deposition of mercury from the air is now known to become rapidly available for methylation and uptake in fish (Harris et al. 2007).

Today, thousands of lakes and rivers worldwide are seriously polluted with methylmercury. The extent of the problem is illustrated by fish advisories in the United States. The U.S. EPA maintains a registry of warnings on fish contamination, where advice is provided about the safety of eating local fish (U.S. EPA 2009). The total number of advisories for mercury has been increasing and, by 2008, it exceeded 4,000. More than 80% of all advisories have been issued, at least in part, because of mercury; they affected > 16 million lake acres and > 1.3 million river miles in 2008 (U.S. EPA 2009).

Although mercury has been thought to be a natural component in the biosphere, compilation of mercury analyses from tissues of Arctic indicator species shows that current-day levels are increased by a factor of about 10 above those present in preindustrial times (Dietz et al. 2009).

## Regulatory Decisions

After the use of mercury fungicides in agriculture had been banned in 1966 in Sweden, a similar ban was instituted in 1970 in the United States, spurred by media reports that mercury-treated grain had been used by a New Mexico farmer to feed his hogs, and that contaminated pork from the farm had entered the market (Davis et al. 1994; D’Itri and D’Itri 1978).

The first safety evaluation of methylmercury in fish took place in Sweden in 1968 and relied on the Japanese data (Landell 1968). Seafood from Japan was thought to contain an average methylmercury concentration of 50 μg/g (measured as mercury). The Swedish experts at the National Institute of Public Health then figured in a safety factor of 10 and another one of 5 to arrive at a safe mercury limit of 1 μg/g for all seafood (Landell 1968). However, some confusion occurred, because the original Japanese data turned out to be based on dry weight concentrations but the Swedish monitoring data used wet weights. Dry weight concentrations are up to five times higher than wet. The calculated limit then should logically apply to dry weight concentrations, or it should be decreased by 80% if applied to wet weight. The error was due to a missed footnote in an English-language report (Landell 1968). Further refinement was published in a detailed risk assessment (Swedish National Institute of Public Health 1971). These experts recommended a safe limit for dietary exposure of 0.4 μg/kg body weight per day, corresponding to a hair mercury concentration of about 6 μg/g. At that level, an adult weighing 70 kg could eat 200 g of fish per week at a mercury concentration of 1 μg/g.

The first international evaluation of methylmercury toxicity (JECFA 1972) recommended a provisional tolerable weekly intake of 200 μg (3.3 μg/kg body weight), that is, virtually the same as the limit proposed in Sweden, after allowing for the difference between daily and weekly intake. In a subsequent review, the International Programme on Chemical Safety (1990) concluded that, based on the data on developmental neurotoxicity from the main Iraqi incident, fetal neurotoxicity might occur when maternal hair mercury concentrations exceed 10–20 μg/g.

In Japan, mercury analyses of fish had already begun in the 1960s, but it was only in 1973 that systematic studies became feasible. A provisionally tolerable limit of 0.4 μg/g (as total mercury, and 0.3 μg/g as methylmercury) was set by the Japanese Ministry of Health and Welfare for fish intended for human consumption (Endo et al. 2005). This limit remains in effect, although it does not apply to tuna, swordfish, and freshwater fish.

In the United States, a limit of 0.5 μg/g was already in use in 1970, when analyses of canned tuna revealed that the limit was exceeded (Mazur 2004). This finding led to a governmental recall of both tuna and swordfish. In 1985, the conundrum of methylmercury accumulation in marine food chains was resolved by increasing the permissible limit to 1 μg/g for the relevant species. The previous recall of mercury-contaminated fish was therefore criticized as a “false alarm” (Mazur 2004).

Within the European Union, a common limit of 0.5 μg/g had been applied to fish in general since 1993, but a few species, such as tuna and swordfish, were allowed to contain up to 1 μg/g. This regulation proved problematic because member states reported mercury concentrations exceeding the 0.5 μg/g limit in many other species. The European Commission therefore decided in 2001 to add all of these species to the list of those that had to comply with the 1 μg/g limit (European Commission 2001). This decision referred to the need for transparency and the need to maintain mercury levels as low as reasonably achievable, while taking into account “physiological reasons” that mercury concentrates in the tissues of certain species more easily than others. However, no assessment of the associated health risks was produced on this occasion, and no advice was offered to the public.

## Widening of Toxicity Risks

Research in the field of developmental neurotoxicity was highly inspired by the observation of dose-dependent effects of environmental lead exposures (Needleman et al. 1979). Adverse effects from methylmercury also seemed to occur as a continuum: the higher the dose, the more severe the illness. Some of the most highly exposed populations were indigenous groups. In Canada, a study of 234 Cree children showed abnormal tendon reflexes associated with mercury concentrations in maternal hair representing their exposure during pregnancy (McKeown-Eyssen et al. 1983). These findings suggested that even slightly increased environmental exposure to methylmercury from fish could lead to adverse effects on nervous system development, just like inorganic lead exposure.

In a cohort-based case–control study of children exposed to methylmercury from marine food carried out in New Zealand, Kjellström et al. (1986, 1989) measured the mercury concentration in the mother’s hair during pregnancy and then examined the children at various ages. They reported delayed brain development in children from mothers with hair mercury concentrations ≥ 6 μg/g. The results were published after peer review by the Swedish Environmental Protection Agency; however, the findings were at first ignored for formal reasons by other regulatory authorities because the report had not appeared in a peer-reviewed scientific journal—although it was eventually published in one after additional statistical analyses were conducted (Crump et al. 1998).

Two large prospective studies were initiated in the mid-1980s. A study of 1,000 children from the Faroe Islands concluded that low-level methylmercury exposure during intrauterine development was associated with deficits in several brain functions in school-age children and that significant associations were apparent well below a maternal hair mercury concentration of 10 μg/g (Grandjean et al. 1997). In contrast, largely nonpositive findings were initially reported in children from a similar study in the Seychelles (Myers et al. 2003). Although statistical analyses showed that the two studies were not in mutual disagreement because of wide confidence limits (Keiding et al. 2003), the apparent disagreement was perceived as a controversy and fueled a debate on uncertainty (Grandjean 1999). Additional longitudinal data later appeared from Japan, Poland, and the United States in support of the Faroe Islands conclusions (Jedrychowski et al. 2006; Lederman et al. 2008; Murata et al. 2006; Oken et al. 2008). Although less weighty, several cross-sectional studies also supported the existence of low-level exposure neurotoxicity (Grandjean et al. 2005).

The reasons for the apparent lack of mercury effects in the Seychelles could be that beneficial nutrients in fish might obliterate or dampen the mercury toxicity (Clarkson and Strain 2003). In recent research from the Seychelles, Strain et al. (2008) reported that cognitive development in children was not associated with either maternal fish intake or methylmercury exposure, when each was considered separately. If maternal fish intake and mercury were included in the statistical analysis at the same time, fish intake was clearly beneficial, whereas mercury had negative effects. Also, in the Faroe Islands, the mercury toxicity became more prominent after adjustment for the beneficial effects of the mother’s fish intake during pregnancy (Budtz-Jorgensen et al. 2007).

## Interpretation of Uncertainty

Because of the apparent disagreement between the two major studies and mercury’s public health implications, in 1998 the U.S. White House called for an international workshop with 30 invited experts, who were asked to critically examine the scientific evidence. These experts emphasized a variety of possible uncertainties and concluded that “there are inadequate data . . . to draw meaningful conclusions at this time” (National Toxicology Program 1998). Despite the possibility that subclinical toxicity could be easily missed and underestimated, the workshop experts were quite optimistic:

Measurement error can impact significantly on both the estimated levels of effect and the decision on the level of exposure at which an effect is detected because of the potential for misclassification. However, the data presented in the workshop suggest that the precision of measurements of methylmercury in hair or cord blood is very good. (National Toxicology Program 1998)

The experts recommended further research.

At the request of the U.S. Congress, a new expert panel was then convened by the National Research Council (NRC) to determine whether an exposure limit of 0.1 μg/kg body weight per day was appropriate, as proposed by the U.S. EPA on the basis of the Iraq data on adverse effects in children after maternal methylmercury exposure during pregnancy (NRC 2000). The committee supported the U.S. EPA limit but recommended that it should be based on the data from the Faroe Islands study. In addition, dose–response data from all three studies (Faroes, New Zealand, Seychelles) combined were in accordance with the Faroes findings.

A few years later, JECFA (2003) reconsidered its original evaluation from 1972. The experts decided to exclude the New Zealand data from consideration and settled on a weekly intake limit of 1.6 μg/kg body weight. In reaching this conclusion, the health benefits of seafood diets were emphasized, along with the need to prevent consumers from being afraid to eat fish because of mercury contamination. The JECFA experts (JECFA 2003) therefore chose a smaller uncertainty factor than did the NRC committee (NRC 2000). Not wanting to take sides in a discussion on key studies and safety factors, the European Food Safety Authority (2004) recognized both exposure limits and concluded that exposure to this food contaminant “should be minimised.” In the United States, federal agencies currently use different exposure limits when dealing with safe human methylmercury exposures from commercially traded fish, fish contaminated from toxic waste, and fish caught by sport fishers (Grandjean 1999). Each limit is supported by a risk assessment that relies on virtually the same evidence.

Because of the beneficial nutrient contents of seafood, two seafood dinners per week are generally recommended as part of a varied diet. A total weekly seafood intake including two fish dinners would represent about 500 g of seafood. The U.S. EPA limit suggests that an adult (weight, 70 kg) should not exceed a mercury intake of 0.1 μg × 7 days × 70 kg body weight, or about 50 μg (U.S. EPA 2001). Therefore, the seafood should contain an average mercury concentration of ≤ 0.1 μg/g. However, current regulations in the United States and the European Union allow up to 10 times as much.

A more accurate risk calculation would need to take into account the masking effects of essential nutrients that promote brain development (Budtz-Jorgensen et al. 2007). Further, the above risk assessments did not take into account the consequences of imprecision in exposure assessments and the implications of misclassification (Budtz-Jorgensen et al. 2003, 2004). Standard statistical analyses assume that exposure biomarkers are measured without error, which is not possible because they are merely proxy indicators of the true dose to the brain. Statistically, any random error will cause an underestimation of the true effect. However, the total imprecision of the cord-blood analysis was much greater than suggested by the laboratory quality control, and the hair mercury analysis was even more imprecise (Budtz-Jorgensen et al. 2004). Dose–response relationships based on the hair mercury concentration therefore significantly underestimated the true mercury effect. Accordingly, the benchmark dose level decreased by about 50% after adjustment for the imprecise exposure data (Budtz-Jorgensen et al. 2004). Thus, the exposure limits estimated by the U.S. EPA and JECFA would need to be halved. Additional imprecision may occur from using nonspecific outcome variables that are affected by other factors.

**Figure 1:**
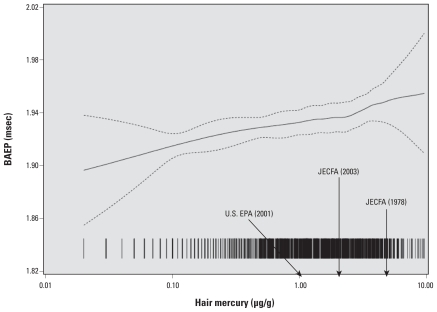
Association between brainstem auditory evoked potential (BAEP) latency (interpeak III–IV at 20 Hz) and dietary exposure to methylmercury (reflected by hair mercury concentration) in 14-year-old Faroese children. Data are from examinations of a Faroese cohort of 878 subjects at 14 years of age. Each vertical line represents one subject, dotted lines indicate the 95% confidence limits, and arrows represent three methylmercury exposure limits. Modified from Murata et al. (2004).

Sophisticated techniques, such as neuro-physiological detection of delayed electrical transmission in the brain, have shown adverse effects at very low mercury exposure levels (Figure 1) (Murata et al. 2004). Data seem to indicate that there may not be an actual threshold for methylmercury toxicity, although the exact cognitive implications of slightly delayed electrical signals in the brain are unclear at this point in time.

The combined evidence led the UNEP to initiate a global assessment project for mercury, and an international agreement on mercury pollution abatement was approved by the member states in 2009 (UNEP 2009). The European Union and the United States have already decided on a ban on mercury exports, and mercury is being phased out in thermometers and scientific instruments (European Union 2007). The time scale in Table 1 suggests that all of these preventive measures followed at a substantial delay after the discovery of environmental health problems, partially because of disagreement about the impact of uncertainties.

## Lessons for Environmental Health Research

The first observed cases of methylmercury poisoning, almost 150 years ago, occurred from incautious experimental practices, and many subsequent poisonings in workers were due to methylmercury inadvertently formed during production processes. However, these early discoveries of the toxicity of methylmercury and its formation during chemical production were subsequently forgotten or disregarded, thereby causing delays in knowledge building and prevention.

The failure to recognize the distinctive clinical features of serious methylmercury poisoning in adults delayed the identification of the etiology of Minamata disease and thus recognition of the full extent of the outbreak. Even when methylmercury had been established as the chemical cause of the disease, strict diagnostic requirements and case definitions assumed that the disease was a characteristic all-or-none phenomenon, thereby excluding less distinctive cases and obscuring the dose–effect relationship.

The first likely cases of developmental methylmercury poisoning were described in 1952 (Engleson and Herner 1952) and subsequently reported from Minamata (Harada 2004; Social Scientific Study Group on Minamata Disease 1999); replication in laboratory animals was published in 1972 (Spyker et al. 1972), and the first prospective population study of prenatal methylmercury toxicity due to contaminated seafood in humans was published in 1986 (Kjellström et al. 1986). However, scientific consensus on prenatal vulnerability was hampered by focusing on uncertainties in the evidence, and international agreement on the need for protection against prenatal exposures was reached only in 2003.

Environmental methylation of mercury in sediment was discovered accidentally because systematic studies of the environmental fate of mercury were not conducted and because initial studies focused only on the total mercury concentration. Recognition of food chain contamination and environmental bioaccumulation of methylmercury was therefore delayed.

A key experiment showing that cats developed the characteristic disease when fed effluent water from a polluting factory was suppressed by the sponsoring company, and the detailed results became available only after a delay of 40 years (Eto et al. 2001). Industry representatives advanced alternative explanations and used the early diversity of scientific opinions for its legal argument that toxicity was not the responsibility of industry, nor could it have been anticipated, thereby causing a substantial delay of remediation, compensation, and prevention.

After the publication of new data on adverse effects of low-level exposures to methylmercury, regulatory agencies requested scientific scrutiny. Expert committees emphasized uncertainties and weaknesses in the available data. Less attention was paid to the question of what could have been known, given the research methods and possibilities, and whether developmental neurotoxicity at low methylmercury doses could be ruled out. The reports also generally ignored that measurement imprecision most likely resulted in an underestimation of the true effects. Instead, more research was recommended. The insistence on solid evidence promoted by polluters and regulatory agencies therefore agreed with a desire among researchers to expand scientific activities in this area. However, the wish to obtain more complete proof had the untoward effect of delaying corrective action.

Although Jun Ui’s critique (D’Itri and D’Itri 1978), given in the introduction of this review, referred to Japanese regulatory agencies themselves being afflicted by signs of methylmercury poisoning, here we suggest that environmental health research, too, has suffered tunnel vision, forgetfulness, lack of coordination, and some of the other symptoms noted in poisoning cases. Like methylmercury poisoning itself, such abnormalities deserve preventative action.

## Figures and Tables

**Table 1:** Important early warnings about and recognition of methylmercury (MeHg) toxicity.

Year(s)	Event	References
1865	First published record of fatal occupational MeHg poisoning	Edwards 1865
1887	First experimental studies on MeHg toxicity	Hepp 1887
1930	Report on organic mercury poisoning in acetaldehyde production workers	Koelsch 1937
1940–1954	Poisoning cases in workers at MeHg fungicide production plants	Franke and Lundgren 1956; Hunter and Russell 1954
1952	First report on developmental MeHg neurotoxicity in two infants	Engleson and Herner 1952
1956	Discovery of a seafood-related disease of unknown origin in Minamata, Japan	SSSGMD 1999
1959	Studies on MeHg toxicity in cats suppressed by the polluting company	Eto et al. 2001
1967	Demonstration of mercury methylation in sediments	Jensen and Jernelov 1967
1968	Official acknowledgment of MeHg as cause of Minamata disease	SSSGMD 1999
1955–1972	Occurrence of poisoning epidemics from use of MeHg-treated seed grain for cooking, and decline in exposed wildlife populations	Bakir et al. 1973; Borg 1969
1972	Experimental study of delayed effects due to developmental neurotoxicity	Spyker et al. 1972
1972	JECFA exposure limit of 3.3 μg/kg per week based on toxicity in adults	JECFA 1972
1973	Report on dose–response relationship in adults from Iraqi data	Bakir et al. 1973
1986	First epidemiology report on adverse effects in children related to maternal fish intake during pregnancy in New Zealand	Kjellström et al. 1986
1997	Confirmation from prospective study in the Faroe Islands on adverse effects in children from MeHg in maternal seafood intake during pregnancy	Grandjean et al. 1997
1998	White House workshop of 30 scientists identifies uncertainties in evidence	NTP 1998
2000	NRC supports exposure limit of 0.1 μg/kg per day	NRC 2000
2003	Updated JECFA exposure limit of 1.6 μg/kg per week	JEFCA 2003
2004	European Union expert committee recommends that exposures be minimized	EFSA 2004
2005	European Union decides on a ban on mercury exports	European Union 2007
2009	International agreement on controlling mercury pollution	UNEP 2009

Abbreviations: EFSA, European Food Safety Authority; JECFA, Joint Expert Committee on Food Additives; NRC, National Research Council; NTP, National Toxicology Program; SSSGMD, Social Scientific Study Group on Minamata Disease.
